# Placental Vascular Calcification and Cardiovascular Health: It Is Time to Determine How Much of Maternal and Offspring Health Is Written in Stone

**DOI:** 10.3389/fphys.2018.01044

**Published:** 2018-08-07

**Authors:** Mary C. Wallingford, Ciara Benson, Nicholas W. Chavkin, Michael T. Chin, Martin G. Frasch

**Affiliations:** ^1^Mother Infant Research Institute, Tufts Medical Center, Boston, MA, United States; ^2^Molecular Cardiology Research Institute, Tufts Medical Center, Boston, MA, United States; ^3^Department of Bioengineering, University of Washington, Seattle, WA, United States; ^4^Yale Cardiovascular Research Center, Yale University School of Medicine, New Haven, CT, United States; ^5^School of Medicine, Division of Cardiovascular Medicine, University of Virginia, Charlottesville, VA, United States; ^6^Department of Obstetrics and Gynecology, University of Washington, Seattle, WA, United States

**Keywords:** biomarkers, cardiovascular health, ectopic calcification, placenta, vascular calcification

## Abstract

Vascular calcification is the deposition of calcium phosphate minerals in vascular tissue. Vascular calcification occurs by both active and passive processes. Extent and tissue-specific patterns of vascular calcification are predictors of cardiovascular morbidity and mortality. The placenta is a highly vascularized organ with specialized vasculature that mediates communication between two circulatory systems. At delivery the placenta often contains calcified tissue and calcification can be considered a marker of viral infection, but the mechanisms, histoanatomical specificity, and pathophysiological significance of placental calcification are poorly understood. In this review, we outline the current understanding of vascular calcification mechanisms, biomedical consequences, and therapeutic interventions in the context of histoanatomical types. We summarize available placental calcification data and clinical grading systems for placental calcification. We report on studies that have examined the association between placental calcification and acute adverse maternal and fetal outcomes. We then review the intersection between placental dysfunction and long-term cardiovascular health, including subsequent occurrence of maternal vascular calcification. Possible maternal phenotypes and trigger mechanisms that may predispose for calcification and cardiovascular disease are discussed. We go on to highlight the potential diagnostic value of placental calcification. Finally, we suggest avenues of research to evaluate placental calcification as a research model for investigating the relationship between placental dysfunction and cardiovascular health, as well as a biomarker for placental dysfunction, adverse clinical outcomes, and increased risk of subsequent maternal and offspring cardiovascular events.

## Introduction to placental calcification

Placental calcification is the deposition of calcium-phosphate minerals in placenta tissue. It is seen in patients with and without placental diseases (Tindall and Scott, 1965). Placental calcification is diagnosed non-invasively by ultrasonographic examination and identification of echogenic foci, and is used as a marker of viral infection (Bailão et al., 2005). Placental calcification is classified by Grannum grading. The Grannum classification system includes grades 0, I, II, and III. Grades 0 placentas display homogenous texture with minimal mineral deposition; on the other end of the spectrum grade III placentas are highly calcified and characterized by echogenic indentations resembling cotyledons (Grannum et al., 1979; Mastrolia et al., 2016). Highly calcified grade III placentas often prompt expedited delivery and have been associated with a higher risk of adverse pregnancy outcome (Chen et al., 2011; Mirza et al., 2017).

**Figure 1:**
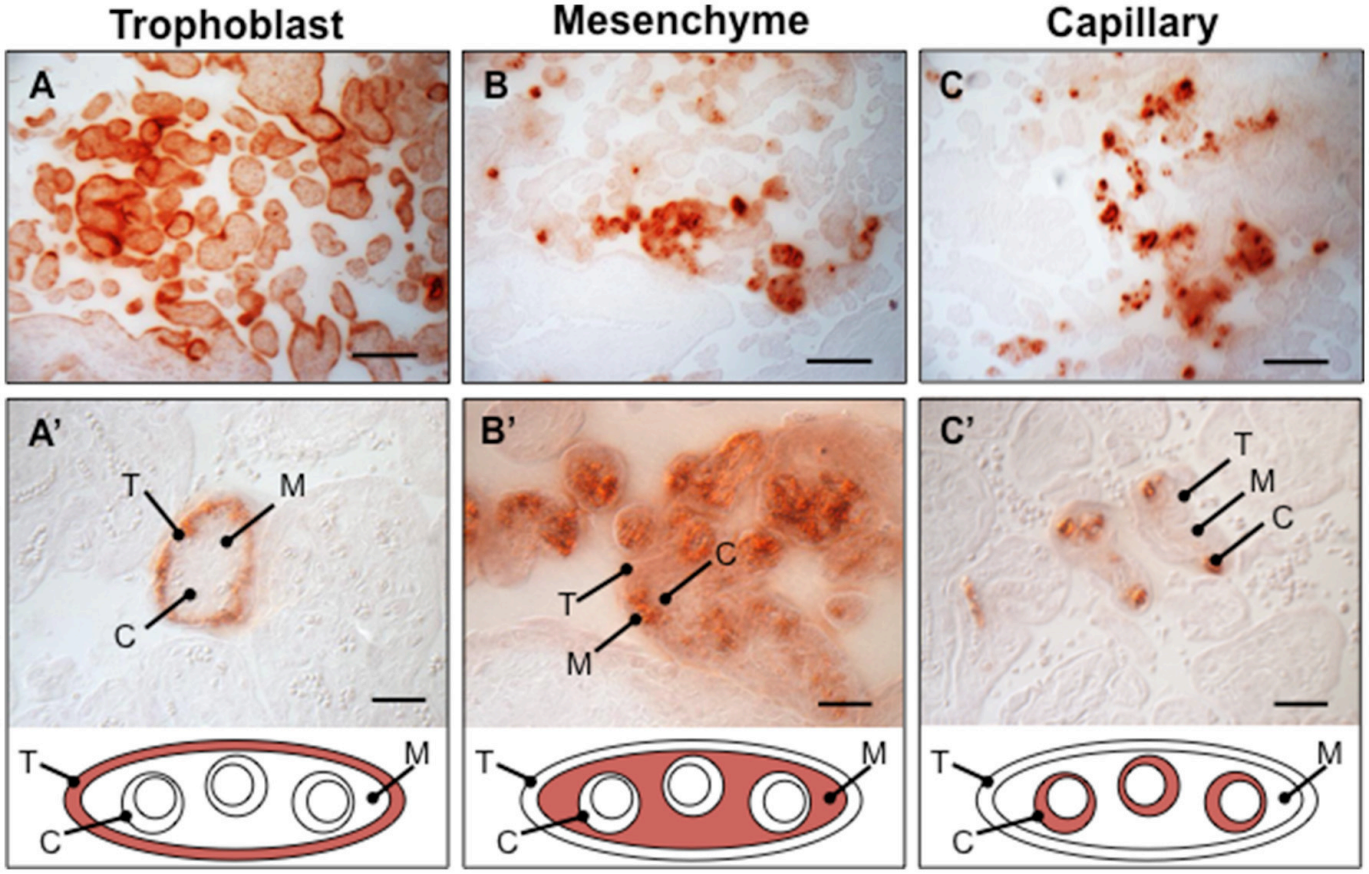
Observed calcium pattems in human placenta villi. Placental biopsies between 28-33week gestation age were paraffin embedded, sectioned, and stained with Alizarin Red (*N* = 16 placentae). Several distinct calcium deposition patterns varying in histoanatomical specificity were identified in chorionic villi, including trophoblast **(A,A')**, mesenchyme **(B,B')**, capillary **(C,C')**, and mixed (not shown). T, trophoblast; M, mesenchyme; C, capillary. Scale bar in **A–C**: 100 μm. Scale bar in D–F: 20 μm.

The physiological ramifications of placental calcification are not known and advanced noninvasive imaging techniques are needed in order to permit accurate assessment of distinct mineral deposition patterns and their impact on placental physiology (Moran et al., 2013). Targeted histoanatomical examination of mineral deposition in chorionic villi reveals multiple distinct microcalcification patterns in human placenta prior to 33 weeks gestation (Figure 1). The Grannum classification system does not provide an adequate mechanism for physicians to use to accurately describe and record this level of complexity. Routine ultrasound image analysis is inadequate for resolving cell-type specific microcalcification patterns. Therefore, a diagnostic approach based exclusively on Grannum grading is not practical for assessment of the incidence and physiological impact of placental calcification.

## Interaction of placental calcification with acute maternal and fetal outcomes

Calcification is commonly observed in placenta, but the incidence and interaction with acute clinical outcomes remains unclear. Clinical research on placental calcification is limited and discordant (Mastrolia et al., 2016). A study comparing normal and high-risk gravidas found that the average grades were similar, but hypertension and intrauterine growth restriction associated with higher grades while diabetes and Rh sensitization associated with lower grades (Hills et al., 1984). A study of 230 term and preterm pregnancies determined that grade III is associated with pregnancy complications, as well as gestational age (Kazzi et al., 1984). Further, (McKenna et al., 2005) examined 802 low-risk patients at 36 weeks and found that grade III placenta helps to predict subsequent development of proteinuric pregnancy-induced hypertension and may help in identifying growth-restricted babies (McKenna et al., 2005). In contrast, (Patterson et al., 1983) found that grade II is associated with lower birth weight and grade III with growth retardation, but neither associated with poor perinatal outcome, maternal hypertension, fetal distress, or perinatal asphyxia (Patterson et al., 1983). A recent meta-analysis by Mirza et al. (2017) supports a positive association between grade III and labor induction, but not fetal distress, Apgar score, neonatal resuscitation, or NICU admission (Mirza et al., 2017). The discordance in study outcomes may relate in part to the crude nature of Grannum grading, which does not provide a mechanism by which to distinguish cell type-specific calcification patterns. Additional sources of discordance may include variance in clinical practices/protocols, population characteristics, sample size, or gestation day.

Timing of scans and calcification onset provide greater context and should be included in assessments of predictive value. A study of 246 placentas within 1 week of delivery found a grade III incidence of 39.4% and no association with altered fetal growth or birth weight (Miller et al., 1988). Vosmar et al. found no correlation between grade III and fetal growth restriction at term and a positive correlation between growth retardation and grade III detected prior to 36 weeks (Vosmar et al., 1989). A study of ~50 patients found no association between perinatal complications and grade III at 37–41 weeks, and identified perinatal complications in 62.5% of patients with grade III prior to 37 weeks (Baeza Valenzuela and García Méndez, 1995). In contrast, 1709 scans examined support that only ~20% of placentas had extensive calcification and grading could not be used to predict postmaturity (Hill et al., 1983). A study of 2000 Thirty-Four and Thirty-Six weeks cases supported a decrease in the risk of perinatal death when grading was known, providing a basis for the reporting of placental grading during the third trimester (Proud and Grant, 1987). A comparison between 31 and 34 week placentas identified increased incidence of intrauterine growth retardation (6.20%), fetal distress (7.8%), and low birth weight (34.37%) with grade III calcification (Chitlange et al., 1990).

Together, these studies suggest that early appearance of calcification may be useful for identifying high-risk cases. Detailed, high-powered cell-type specific temporal analysis across gestation is needed to provide a clear picture of calcification onset and progression. In turn, this research would hold great potential to inform the development of a revised classification system that may improve clinical value of placental grading.

## Intersection between placental calcification and long-term cardiovascular health

Placental health may be an indicator of maternal cardiovascular disease risk. Preeclampsia is a maternal hypertensive disorder starting after 26 weeks of pregnancy and accompanying increased proteinuria. Preeclampsia is likely caused by placental dysfunction and the only cure for preeclampsia is delivery of the placenta. Hypertension-associated risks continue postpartum, and include stroke, congestive heart failure, and renal failure (Pauli and Repke, 2015). Six months postpartum, mothers have increased levels of coagulation, complement activation, and cardiovascular disease risk markers (Murphy et al., 2015). Mothers with a history of preeclampsia are at increased risk of cardiovascular disease 10 to 15 years after pregnancy, including risk factor increases in hypertension (3.70), ischemic heart disease (2.16), myocardial infarction (2.00), venous thromboembolism (1.79), and stroke (1.81) (Smith et al., 2001; Bellamy et al., 2007; Ahmed et al., 2014). Cardiovascular disease risk continues 30 years after pregnancy (2.14 hazard ratio) (Mongraw-Chaffin et al., 2010), along with increased risk of hypertension-associated neuropathologies, with an almost 3-fold increase in presence of cognitive impairment 35–40 years after pregnancy (Fields et al., 2017). Additionally, patients with preeclampsia and late-life hypertension showed decreased brain volume and decreased gray matter, suggesting associations between preeclampsia, cardiovascular disease and cerebrovascular diseases (Raman et al., 2017). A mechanistic study found evidence of subclinical atherosclerosis in postmenopausal women who had a history of preeclampsia (Jayachandran et al., 2017). Finally, coronary artery calcification was 3.5 times more likely in patients with a history of preeclampsia, and 2.6 times more likely when adjusting for hypertension (White et al., 2016).

There are common risk factors for preeclampsia and vascular calcification, including diabetes, chronic hypertension, obesity, renal disease, and age (Duckitt and Harrington, 2005; Dayan et al., 2015). There may be common causes or interacting etiologies. A population-based study of Rochester Epidemiology Project postmenopausal women identified preeclampsia as a risk factor for coronary artery calcification and revealed an association of coronary artery calcification and microvesicles positive for tissue factor and stem/progenitor cell antigen CD117 in women with a history of preeclampsia and elevated metabolic risk profile (Miller et al., 2016). An ongoing study aims to evaluate the association of reproductive disorders, including preeclampsia, with cardiovascular risk and coronary artery calcium scores (Zoet et al., 2017).

No studies have directly assessed the intersection of placental calcification with long-term risk of vascular calcification or with onset and progression of preeclampsia. The relationship between placental architecture and placental disease is not completely clear (Mastrolia et al., 2016). Further investigation is needed to delineate associations between preeclampsia, placental calcification, and vascular calcification in order to evaluate the potential diagnostic value of placental calcification in both acute and long-term cardiovascular health.

## Avenues of research to evaluate the diagnostic value of placental calcification

New diagnostic tools are needed to evaluate placental calcification. The diagnostic value of placental calcification, including onset, abundance, and cell-type specificity, is currently unknown. We suggest that advanced, standardized diagnostic mechanisms are needed for nuanced assessment of onset and cell-type specificity of placental calcification during pregnancy. We also suggest that placenta calcification be incorporated into studies that investigate the interaction of placental health and with both acute and long-term maternal and fetal outcomes.

There is currently a knowledge gap between placental, maternal, and fetal health. Heart fitness is influenced by multiple prenatal cues, including hemodynamic pressure, growth factor levels, and availability of oxygen and essential nutrients, all of which are influenced by the placenta. Heart rate variation analyses support that both prenatal stress and preeclampsia affect the autonomic nervous system (Voss et al., 2006). Children exposed to preeclampsia have increased risk of hypertension and BMI (Davis et al., 2012), and are twice as likely to suffer stroke (Kajantie et al., 2009). Hence, primary chronic diseases affecting western societies may originate in prenatal life. Well-controlled clinical evaluation studies and innovative application of biological models are needed to close this knowledge gap between placental, maternal, and fetal health and advance the clinical interpretation of placental disease.

## Calcific potential of the placenta

Placental calcification mechanisms may shed light on common etiologies with other forms of ectopic calcification. Ectopic calcification can form by metastatic, dystrophic, or physiologic mechanisms. Metastatic calcification affects healthy tissue and is associated with abnormal levels of calcium and phosphate (Karwowski et al., 2012). Dystrophic calcification occurs in dead or necrotic tissue. Physiologic calcification is observed in bone formation (Frink, 2002). Molecular vascular calcification mechanisms are a complex intersection of regulatory pathways, including adenosine signaling, osteochondrogenesis, inflammation, endocrine pathways, hypoxia, malnutrition, autophagy, phosphate transport, and phosphate signaling. Please see these recent reviews for current detail on cardiovascular calcification mechanisms (Wu et al., 2013; Hortells et al., 2018; Zazzeroni et al., 2018).

Mechanisms that regulate placental calcification have not been fully defined. Examination of mineral chemical content and BMP levels indicated a metastatic calcification mechanism (Poggi et al., 2001). The activation of programmed cell death pathways and presence of necrotic tissue suggests dystrophic calcification is likely to contribute to placental calcification. Of note, medial calcification associated with chronic kidney disease was initially described as a dystrophic process, but has since been supported as an active, regulated process. It remains largely unexamined whether physiological calcification mechanisms contribute to placental calcification.

Expression of osteochondrogenic genes in vessels has been observed in in preclinical and clinical studies (Speer et al., 2009; Ho and Shanahan, 2016; Lindman et al., 2016; Mosch et al., 2017; Panh et al., 2017). Expression of osteochondrogenic genes has also been detected in placenta, including adrenomedullin, alkaline phosphatase, BMPs, osteocalcin, osteopontin, osteoprotegrin, placental growth factor, Slc20a1, and Slc20a2 (Poggi et al., 2001; Johnson et al., 2003; Lenhart and Caron, 2012; Tannetta et al., 2013; Chen and Karumanchi, 2015; Yang et al., 2015; Wallingford et al., 2016b). The Slc20a2 knockout mouse develops ectopic calcification in cerebral vasculature and placenta, providing genetic context and suggesting that impaired phosphate transport may be a cause of calcification (Wallingford et al., 2016a,b). Phosphorus is required in abundance for fetal growth, metabolism, and skeletal development, and placenta is exposed to high levels of phosphorus during pregnancy. At birth serum phosphorus levels are 4.37 ± 1.62 mg/dl in maternal venous, 5.96 ± 1.26 mg/dl in umbilical venous, 5.83 ± 1.24 mg/dl in umbilical arterial, and 6.53 ± 148 mg/dl in newborn capillary blood (Schauberger and Pitkin, 1979). Inorganic phosphate may also act as a signaling molecule that influences osteoblastic gene expression and mineral deposition (Chavkin et al., 2015; Panh et al., 2017).

Placenta cells have direct and indirect osteogenic potential *in vitro*. Mesenchymal placenta-derived adherent cells (PDACs) stimulate endogenous osteoblastogenesis (Li et al., 2011). Human fetal early chorionic stem cells (e-CSC) demonstrate bone repair in a murine osteogenesis imperfecta model, reducing fractures and increasing bone volume, hypertrophic chondrocyte number, and ossification gene expression (Jones et al., 2014). Amniotic epithelial placental cells demonstrate positive control of bone regeneration *in vivo* (Barboni et al., 2013). In support of differentiation, mesenchymal cells from the placenta (PMSC) display endogenous osteogenic potential in 2D cell culture (Takahashi et al., 2004) and osteogenic composite grafts (Jin et al., 2014; Wang et al., 2014).

## Candidate pathways to placental calcification

Adenosine signaling provides a mechanistic intersection of calcification and preeclampsia pathways. Impaired adenosine signaling promotes vascular calcification through an imbalance of pro- and anti-calcific molecules (Lindman et al., 2016). NT5E is causative for the calcification disorder ACDC (Markello et al., 2011; St Hilaire et al., 2011). CD73 (the protein product of NT5E) is upregulated in preeclampsia biopsies and PE-IgG animal models (Iriyama et al., 2015; Salsoso et al., 2017). ADA mice display elevated adenosine, fetal growth restriction, and preeclampsia-like phenotypes, and these can be partially rescued by ADORA2B loss (Iriyama et al., 2015; Salsoso et al., 2017). Adenosine acts in a paracrine manner to promote osteogenic differentiation of human mesenchymal stem cells (Shih et al., 2014). Interactions with alkaline phosphatase, osteoprotegrin, and calcitonin have also been observed in preeclampsia (Lenhart and Caron, 2012; Tannetta et al., 2013; Lenhart et al., 2014; Yang et al., 2015).

Hormones are also poised to regulate placental calcification. Hormones with known pro- or anti-calcific functions act at the maternal-fetal interface, where they influence minerals essential for embryonic growth and development, including vitamin D, parathyroid hormone, parathyroid hormone–related protein, calcitonin, and Fgf23 (Mitchell and Jüppner, 2010; Ohata et al., 2016). Hypovitaminosis D during pregnancy correlates with preeclampsia and gestational diabetes mellitus (Shin et al., 2010) and may pose risk for osteoporosis and pregnancy-related complications later in life (Sofi et al., 2017). Vitamin D receptor (VDR) loss results in hypocalcaemia, rickets, osteomalacia, lower cortical bone density, and increased numbers of osteoclasts (Rummens et al., 2003). Placental VDR is decreased in fetal growth restriction and has been suggested as a therapeutic candidate (Murthi et al., 2016). Supplemental maternal calcium can rescue mineralization in VDR models and improve fetal bone growth (Rummens et al., 2003; Young et al., 2012) and active vitamin D metabolites increase the invasive capacity of extravillous trophoblast (Chan et al., 2015). Fgf23 was shown to regulate vitamin D metabolism in placenta of an X-linked hypophosphatemic rickets/osteomalacia model, but was dispensable for maternal-fetal phosphorus homeostasis and bone growth (Ma et al., 2014; Ohata et al., 2014).

Stress pathways should also be considered as potential mediators of placental calcification. Prenatal stress is experienced by ~20% of pregnant women (Vidal et al., 2014) and is linked to systemic and organ-specific temporally sensitive programming effects, such as epigenetic programming of the hypothalamic-pituitary-adrenal axis and the autonomic nervous system (Frasch et al., 2017a). Pregnancy stress alters uterine blood flow, placental catecholamine, and lactate clearance (Dreiling et al., 2017). Pre-conceptual or intra-gestational stress may also increase cerebral and placental expression of cortisol releasing hormone, stimulating fetal cortisol and adrenocorticotropic hormone, signaling premature maturation of fetal tissue (Horan et al., 2000; Moog et al., 2016). Chronically high levels of circulating stress hormones and lactate may have an impact on placental health, mediating the impact of prenatal stress on preterm birth and gestational diabetes (Shapiro et al., 2013). Lastly, levels of placental O-linked-N-acetylglucosamine transferase (OGT) and O-GlcNAcylation alter with prenatal stress (Howerton et al., 2013). OGT also promotes vascular calcification in diabetes mellitus and promotes osteoblast differentiation (Heath et al., 2014; Koyama and Kamemura, 2015).

Overall, little is known about the interaction between prenatal stress and placental calcification. Associations have been found between vascular calcification and the autonomic tone measured by heart rate variability (Khalaf et al., 2015). The pregnant sheep model of chronic stress (Rakers et al., 2015, 2016) in conjunction with a quantifiable placentome physiology (Vonnahme et al., 2006) represents a model of placental response to stress that may shed light on the interaction of stress and calcification (Frasch et al., 2017b). We speculate that patterns of placental calcification act as a memory of prenatal stress exposure. Linking such patterns to temporal physiological profiles of heart rate variability and autonomic nervous system activity may aid in identifying women and offspring at high risk for cardiovascular and metabolic diseases in later life.

## Synthesis

**Figure 2:**
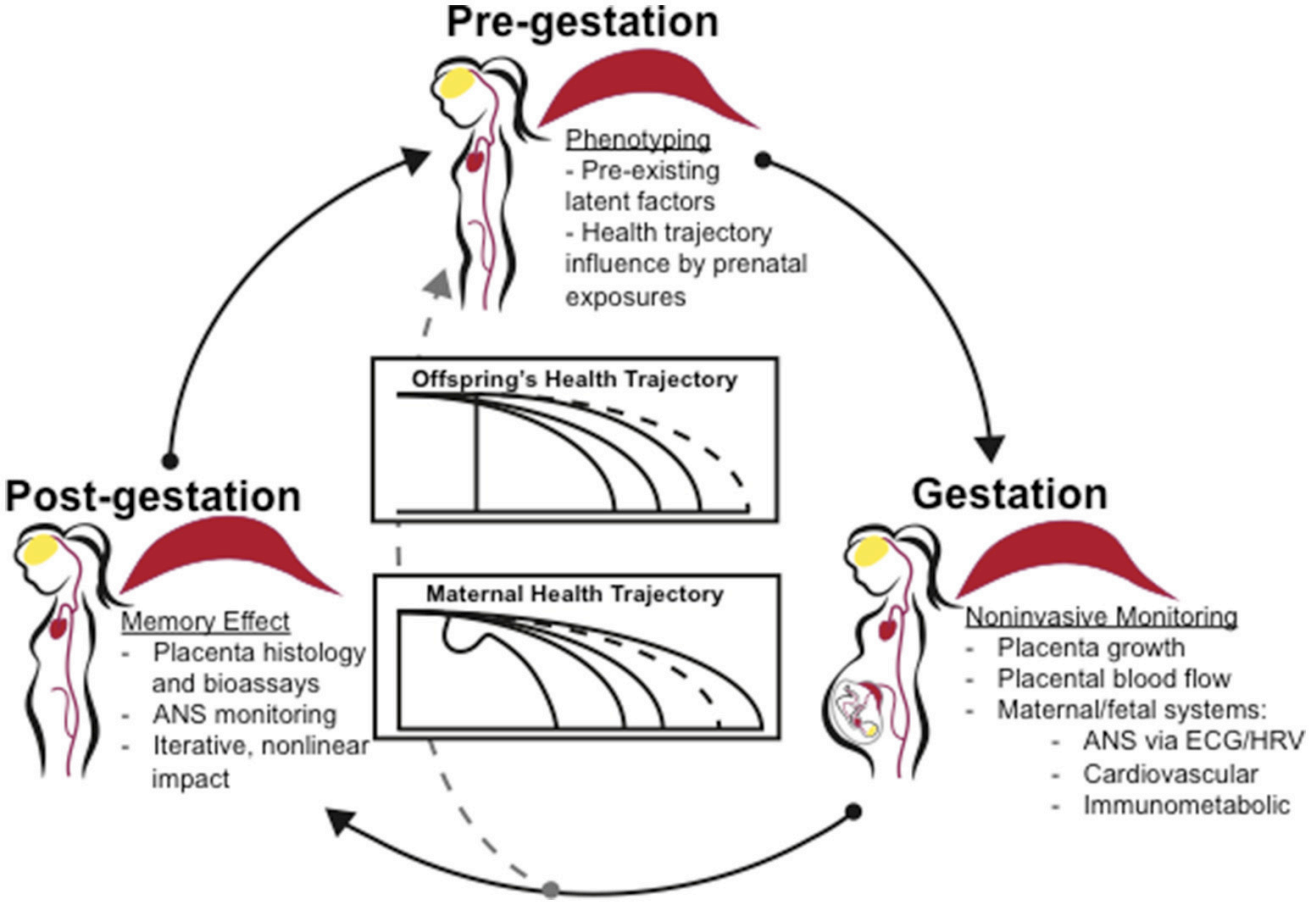
Clinical application of the concept “placenta as a programming agent”. Individual phenotypes and health trajectories may be influenced by both an individual's prenatal development as well as pregnancy complications. Clinical assessment of individual phenotypes by noninvasive biomarkers and with respect to developmental trends may shed light on disease prevention, improved treatment options and disease management. ANS activity, monitored noninvasively via EGG, may serve as proxy for maternal, fetal, and offspring's memory of adaptations to the pregnancy indicating health trajectories. Combined with assessment of placenta and latent factors including cardiovascular and immunometabolic status, we may gain a more individualized prognostic and diagnostic view of pregnancy course with respect to maternal and offspring's health outcomes. ANS, autonomic nervous system; EGG, electrocardiogram; HRV, heart rate variability.

There is a clear relationship between preeclampsia and cardiovascular disease risk later in life, but the causes and ramifications of placental calcification in placental dysfunction and preeclampsia remain unknown. Placental calcification may be a physiological downstream response common to a number of triggers and formed by both common and distinct mechanisms. Maternal phenotypes and trigger mechanisms may predispose for both placental calcification and cardiovascular disease. Successive pregnancies or exposure to mineral imbalance or endothelial dysfunction may amplify risk. We attempt to capture the iterative, nonlinear nature of these interactions in Figure 2. We suggest that individual health trajectories are influenced by both prenatal development and pregnancy complications. Autonomic nervous system activity, monitored noninvasively via electrocardiogram-derived heart rate variability measures, may serve as proxy for maternal, fetal, and offspring memory of adaptations to pregnancy indicating health trajectories. Combined with assessment of placental calcification and latent factors including cardiovascular and immunometabolic status, we may attain a more individualized prognostic and diagnostic view of pregnancy course with respect to maternal and offspring health outcomes.

The evidence suggests that placental calcification may be linked to inflammation, gestational cardiovascular symptoms, aging, and multiple molecular pathways, including adenosine, OGT, and CRH signaling. Placental calcification may serve as an indicator of multi-hit exposures to infection, hypoxia, prenatal or systemic stress, thereby displaying a memory effect (Figure 2). The placenta is a highly vascularized organ, and it is likely that other mechanisms common to vascular calcification are involved.

The placenta is an organ that rapidly grows and dies. It is also the only organ in the body that forms from tissue derived from two individuals. Thus, it provides a model in which rapid vascular, immune, apoptotic, and other intercellular and intracellular processes can potentially be observed *in vivo* and with patient-specific profiles ideal for precision medicine. We suggest placental calcification as a research model for investigating the relationship between placental dysfunction and cardiovascular health, as well as a biomarker for placental dysfunction and adverse clinical outcomes. Placental calcification may reveal underlying predisposition to vascular calcification. Placental calcification onset and timing may better predict postnatal risks for cardiovascular disease in mother and/or offspring (Figure 2). Human case-control and prospective observational studies and tractable, physiological animal models are greatly needed to test proposed interactions of placental calcification and long-term maternal and offspring cardiovascular health.

## Author contributions

MW provided the original idea for manuscript, wrote the abstract, worked with MF on article premise, conducted literature searches, synthesized content provided by the other authors, wrote several sections of the manuscript, collected the data presented in Figure 1, designed figures, formatted references, and edited the manuscript. CB drafted content and edited manuscript. NC conducted an independent literature search, drafted content, and reviewed the manuscript. MC provided suggestions and feedback, and reviewed the manuscript. MF worked with MW on article premise, drafted the synthesis, reviewed and edited the manuscript, and contributed direction and edits for Figure 2.

### Conflict of interest statement

The authors declare that the research was conducted in the absence of any commercial or financial relationships that could be construed as a potential conflict of interest.
